# Symptoms and health experience in irritable bowel syndrome with focus on men

**DOI:** 10.1111/nmo.14430

**Published:** 2022-09-08

**Authors:** Tetyana Bureychak, Åshild Faresjö, Jenny Sjödahl, Anna‐Karin Norlin, Susanna Walter

**Affiliations:** ^1^ Division of Inflammation and Infection, Department of Biomedical and Clinical Sciences (BKV) Linköping University Linköping Sweden; ^2^ Division of Society and Health, Department of Health, Medicine and Caring Sciences (HMV) Linköping University Linköping Sweden; ^3^ Department of Gastroenterology, University Hospital Linköping County Council of Östergötland Linköping Sweden; ^4^ Department of Health, Medicine and Caring Sciences (HMV), Division of Prevention, Rehabilitation and Community Medicine Linköping University Linköping Sweden

**Keywords:** gastrointestinal symptoms, gender, irritable bowel syndrome, men, sex, socioeconomic

## Abstract

**Background:**

Irritable bowel syndrome (IBS) is a disorder with a predominance in women; IBS in men is less studied. The present study evaluated symptoms as well as health and social experiences of men with IBS.

**Methods:**

This cross‐sectional study included 293 patients with IBS (64 men) and 363 non‐IBS controls (62 men). Gastrointestinal symptom diaries were filled in prospectively, and data on comorbidities and healthcare‐seeking behavior were assessed by questionnaires. Men with IBS were compared with men without IBS and women with IBS.

**Key results:**

Compared with women with IBS, men with IBS had fewer contacts with the healthcare system, fewer psychiatric comorbidities, fewer sleeping problems, and less chronic pain. Urgency to defecate and nausea were less common, and stool frequency was higher in men with IBS. There was no difference between men with and without IBS in terms of educational level, satisfaction with household economy, or living with a partner. In contrast, women with IBS more often lived alone, were more often dissatisfied with household economy, and had a lower educational level than women without IBS. Men with IBS had the same proportion of full‐time employment as men without IBS but in contrast, the proportion of women with IBS in full‐time employment was only 34%, compared to 50% of the women without IBS.

**Conclusion and inferences:**

The present study improves the understanding of men's experiences of IBS and suggests that sex and gender may be integrated into the biopsychosocial model of IBS.

## Graphical abstract

Irritable bowel syndrome (IBS) is a functional bowel disorder with a predominance in women; IBS in men is less studied. The present study improves the understanding of men's experience of IBS.

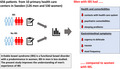

**Key Points**

Despite that the differences between men and women in terms of IBS key gastrointestinal symptoms are relatively small, men with IBS have

- fewer psychiatric conditions
- fewer sleeping problems
- less chronic pain
- fewer contacts with the healthcare system
- better employment status
- higher satisfaction with household economy

## 1 INTRODUCTION

Irritable bowel syndrome (IBS) is one of the most common disorders of gut–brain interaction (DGBI), with a global prevalence around 5%–10%.^1^, ^2^ IBS is characterized by chronic abdominal pain associated with bowel disturbances in the absence of detectable organic disease, and the diagnosis is based on symptom criteria.^3^ One of the most striking discrepancies in terms of IBS is overrepresentation of female patients. A recently published epidemiological study found that IBS was globally present in 5.2% of women and 2.9% of men.^1^ Differences in male and female IBS prevalence are known to be caused by multiple factors including biological, psychological, and environmental components, as well as access to health care and sociocultural differences in response to pain and changes in bowel pattern.^4^, ^5^ Additionally, it is still not clear if some of the reported sex differences in IBS patients may be due to sex‐based differences in health that carry over into the patient population.^4^ Moreover, due to the female predominance, many research studies have insufficient numbers of male participants, which may partly explain why we do not fully understand the reason for the sex differences in IBS.^4^, ^5^ Studies of men with IBS are relatively uncommon, which can probably also limit the possibility to reach men as a patient group. Some of the few studies of men's experience of IBS have analyzed the role of male sex hormones in bowel symptoms in young men with IBS,^6^ brain response to bowel balloon distention in men,^7^ psychological features and relation to gender roles of men with IBS,^8^, ^9^ or comparisons between men's and women's IBS experience and symptomology.^5^, ^10^, ^11^, ^12^, ^13^

Irritable bowel syndrome represents itself in a fairly distinct symptom profile in men and women. One of the common differences is a higher prevalence of diarrhea (IBS‐D) in men and a higher prevalence of constipation in women (IBS‐C).^5^, ^14^, ^15^ Men tend to report lower symptom burden, less bloating and lower abdominal pain duration and intensity, compared to women.^5^, ^13^, ^16^ However, the data on higher stool frequency and lower abdominal pain severity and frequency in men are inconsistent, as some research shows no differences in these symptoms between men and women.^11^ Although statistically significant, sex differences in stool consistency, bloating, and total somatic burden are small, which makes their clinical significance uncertain.^11^ Men are less likely than women to seek health care for additional GI disorders such as globus sensation, dysphagia, constipation, fecal incontinence, and pelvic floor dysfunctions.^14^, ^15^ In addition, men seek health care for extra‐intestinal symptoms, such as migraine headache, fibromyalgia, joint pain, muscle pain, cystitis, and chronic pelvic pain, less often than women.^14^, ^15^ The reasons for these differences in abdominal and extra‐abdominal symptoms in men and women are poorly explored, and they can depend on men's lower symptom burden or men's avoidance of seeking health care for their symptoms, or a combination of both.

In this study, we examine IBS symptoms and psychosocial factors through a sex/gender perspective, and discuss potential reasons for the lower prevalence of IBS in men. We define sex as biological distinctions between females and males, which are based on differences in chromosomes, gene expressions, sex hormones, and reproductive and sexual anatomy. Most often sex is dichotomized into categories of male and female, although sometimes this distinction is hard to make. Gender may or may not be associated with sex. Gender is both a structural characteristic and individual level experience.^17^ On the one hand, it refers to social and cultural expectations, beliefs, and norms associated with being a woman and a man, that is, femininity and masculinity. On the other hand, gender is also an individual identity–social roles with which individuals identify themselves, that is, learned, embodied, and actively constructed femininities and masculinities. Genders and gender identities are dynamic and vary over the individual's life span, and in historical as well as cultural perspectives. Gender is discussed in this article as a *social construct*, that is, as being contextualized and recurrently created though social interactions rather than biologically determined.^18^

It is important to note that although sex and gender are distinct categories, differentiation between biological (sex) and sociocultural (gender) influences on IBS is not always definite, and they might overlap. One example of this is men's and women's perception of symptoms and treatment‐seeking behavior, which can be influenced by both biological factors, such as hormones, differences in visceral sensitivity, and social factors, such as health beliefs, self‐assessment of health status, economic factors, different patterns of responsibilities, and symptoms' impact on everyday activities.^12^ In this paper, we adhere to the terms “men” and “women,” which in this case include both the biological and social/cultural components of personal experience.

The aim was to analyze experiences of men and women with and without IBS through a biopsychosocial perspective. We compared social experiences of men and women with IBS with regard to partnership status, level of education, employment, and household economy. We examined sex/gender differences in terms of psychosocial factors such as experience of health and illness and agency in sustaining health and healthcare‐seeking behavior. Moreover, we studied comorbidity profiles and measured IBS symptoms with both questionnaire and gastrointestinal diary in men and women with and without IBS.

## 2 MATERIAL AND METHODS

### 2.1 Participants and procedure

Recruitment was conducted in 10 primary healthcare centers (PHC) in Östergötland County in the southeast of Sweden between 2010 and 2014 and has been described in more detail previously.^19^, ^20^ These PHCs are responsible for the primary care of a population of approximately 140,000, and they are considered to be a good representation of variation in healthcare problems and sociocultural background of population. A total of 795 patients over 18 years of age with a clinical IBS diagnosis and twice as many controls were invited to participate in the study. The participants were identified based on medical records from PHC and invited by mail. The inclusion of IBS cases was based on a clinical diagnosis of IBS, which was defined according to Rome III criteria (standard at the time), and the presence of IBS symptoms in the previous 2 years. The control group consisted of other patients at these healthcare centers with a similar age and sex distribution, although not matched 1:1, who sought care for other less serious complaints not associated with gastrointestinal (GI) symptoms (i.e., not malignancies or other life‐threatening conditions), and with no GI diagnoses found in the patient register for the previous 2 years.

During their appointment at the PHC, the participants were asked to fill in a GI symptom diary and a form, which included a Rome III questionnaire and a questionnaire for demographic data, general health questions, and questions on perceived health which were derived from the Swedish central government authority for official statistics (Swedish Living Conditions Survey of Welfare and Health). Further questions were asked about medications as well as comorbidity and healthcare utilization. The participants returned their completed forms and GI diaries in a prepaid envelope. This study included only those participants who returned both the completed form and the GI diary, which was a total of 293 patients with IBS and 363 controls.

The question on sex/gender in the questionnaire was formulated as “I am … man/woman,” where the participant could choose the most suitable option. Because the Swedish language has the same words for male and man, and for female and woman, it is difficult to draw conclusions as to whether the respondents identified themselves by sex or by gender. Unfortunately, this question did not include an option for people who refuse binary definitions or cannot choose any of the two options provided.

### 2.2 Measures

#### 2.2.1 Questionnaires

The questionnaire derived from the Swedish Living Conditions Survey of Welfare and Health by the Swedish central government authority for official statistics was used to measure health issues of the participants. It included a question on daily activities to maintain good health with specification and two five‐point scale questions on health rating and rating of health importance. The answers to the latter two were dichotomized into good/bad and important/not important for further analysis. The participants were asked to report which comorbidities they had and how often they had contacted health care during the last year.

#### 2.2.2 The GI symptom diary

The GI symptom diaries were filled in prospectively for 2 weeks. The participants recorded their GI symptoms using validated diary cards.^21^, ^22^ They indicated meal ingestion, symptom occurrence and duration (bloating and nausea), occurrence, duration, intensity and localization of abdominal pain, and several bowel habit items (frequency, Bristol stool form scale, sense of urgency, straining, and sense of complete evacuation). Each of these items was recorded on an hourly basis, 24 h a day for every day of the diary.

### 2.3 Statistical analysis

The results are presented as a median for continuous data with non‐normal distribution and frequency count and percentage for ordinal data. Normality distribution of data was tested with the Shapiro–Wilk test. Significance of association was measured by chi‐square test for categorical variables. Mann–Whitney *U*‐test was used for group comparisons of not normally distributed interval data. Statistical significance was accepted at a level lower than 5% (*p* < 0.05) for both tests.

### 2.4 Ethics approval and consent to participate

All procedures for this study were approved by the Regional Ethical Research Committee at Linköping University (Ref. no. M41‐09). Written informed consent was obtained from all participants.

## 3 RESULTS

### 3.1 Sex/gender differences in social profiles

For baseline social characteristics of the participants, see Table 1.

**TABLE 1:** Baseline social characteristics of the participants

Variable	Men with IBS	Men without IBS	Women with IBS	Women without IBS	Men with IBS vs. men without IBS, *p*	Men with IBS vs. women with IBS, *p*	Women with IBS vs. women without IBS, *p*
Age, median [IQR]	49 [24–70]	55 [22–66]	50 [19–77]	54 [22–67]	0.107	0.928	0.002
Born in Sweden [*n*, %]	56 [88%]	56 [90%]	209 [92%]	280 [93%]	0.614	0.309	0.559
Partnership status [*n*, %]							
Living with a partner	47 [73%]	51 [82%]	157 [69%]	247 [82%]	0.234	0.481	<0.001
Living alone	17 [27%]	11 [18%]	71 [31%]	53 [18%]			
Education level [*n*, %]							
Primary	17 [27%]	7 [11%]	35 [15%]	24 [8%]	0.090	0.082	0.007
Secondary	22 [34%]	27 [44%]	104 [46%]	126 [42%]			
Tertiary	25 [39%]	28 [45%]	89 [39%]	149 [50%]			
Employment [*n*, %]							
Full time employed	40 [63%]	38 [61%]	77 [34%]	149 [50%]	0.084	<0.001	0.001
Part time employed	5 [8%]	4 [7%]	65 [29%]	75 [25%]			
Other	16 [25%]	17 [27%]	79 [35%]	66 [22%]			
Unemployed	2 [5%]	3 [5%]	6 [3%]	11 [4%]			
Satisfaction with household economy [*n*, %]							
Very satisfied	24 [38%]	27 [44%]	48 [21%]	92 [31%]	0.307	0.018	<0.001
Quite satisfied	28 [44%]	21 [34%]	98 [43%]	149 [50%]			
Neither satisfied nor dissatisfied	8 [13%]	8 [13%]	50 [22%]	49 [16%]			
Rather dissatisfied	1 [2%]	5 [8%]	22 [10%]	10 [3%]			
Very dissatisfied	3 [5%]	1 [2%]	10 [4%]	0 [0%]			

*Note:* The category “other” included students, pensioners regardless of reason, persons on a long‐time sick leave and those receiving medical insurance.

The total sample consisted of 656 participants: 126 men and 530 women. There were 293 patients with IBS (64 men) and 363 controls (62 men). The participants with IBS were slightly younger than those without IBS. The median age of IBS patients was 49 years for men and 50 years for women. The median age of controls was 55 years for men and 54 years for women. Most of the participants, both men and women, were of middle and older age, and 92% of the participants were born in Sweden. There was no significant difference between men with and without IBS, men and women with IBS, women with and without IBS in terms of origin (*p* > 0.05 in all group comparisons).

The majority of the participants were in relationships (around 70% and higher), that is, living with a partner and/or being married. Men and women with IBS more commonly lived alone. However, while there was no significant difference between men with and without IBS, women with IBS lived alone more often than women without IBS (*p* < 0.001).

The level of education was generally higher in people without IBS, that is, there were more people with secondary and tertiary education in both groups—men and women without IBS. The difference in the level of education between men with and without IBS and between men and women with IBS was not statistically significant (*p* = 0.09 and *p* = 0.08, respectively). At the same time, comparison of women with and without IBS revealed a statistically significant difference in their level of education (*p* = 0.007).

Analysis of employment status revealed that men regardless of IBS diagnosis more often than women had full‐time employment. Sixty‐three percent of the men with IBS compared to 34% of the women with IBS were employed full‐time (*p* < 0.001). Interestingly, the proportion of men with full‐time employment was very similar in those with an IBS diagnosis (63%) and those without (61%). This difference was much larger among women: 34% with IBS and 50% without (*p* = 0.001). Analysis of employment factors that might be influenced by the IBS diagnosis, that is, long‐term sick leave, early retirement, and receiving medical insurance, revealed no statistically significant differences between men and women with or without diagnosis of IBS.

Comparison of satisfaction with household economy between men with and without IBS was not statistically significant different (*p* = 0.307). In contrast, women with IBS assessed their household economy as poorer compared to women without IBS (*p* < 0.001). In addition, men with IBS tended to be more satisfied with their household economy than women with IBS (*p* = 0.018).

### 3.2 Sex/gender differences in health and comorbidities

A summary of sex/gender differences in health and disease status of the participants is presented in Table 2.

**TABLE 2:** Sex/gender differences in health/disease status

Variable	Men with IBS [*n*, %]	Men without IBS [*n*, %]	Women with IBS [*n*, %]	Women without IBS [*n*, %]	Men with IBS vs. men without IBS, *p*	Men with IBS vs. women with IBS, *p*	Women with IBS vs. women without IBS, *p*
Health rate							
Good	39 [61%]	53 [86%]	141 [62%]	275 [92%]	0.002	0.927	<0.001
Bad	25 [39%]	9 [15%]	88 [38%]	24 [8%]			
Health significance							
Important	61 [95%]	59 [95%]	223 [98%]	294 [98%]	0.968	0.280	0.919
Not important	3 [5%]	3 [5%]	5 [2%]	7 [2%]			
Active actions as to health improvement							
Yes, absolutely	36 [56%]	30 [48%]	108 [47%]	165 [55%]	0.659	0.037	0.119
To some extent	23 [36%]	27 [44%]	115 [50%]	124 [41%]			
No, nothing special	5 [8%]	5 [8%]	6 [3%]	11 [4%]			
Contact with health care during the last 12 months	47 [73%]	45 [73%]	202 [90%]	213 [71%]	0.914	0.001	<0.001
Comorbidities							
Psychiatric disorders	23 [37%]	15 [28%]	118 [52%]	74 [30%]	0.151	0.027	<0.001
Sleep disorder	30 [48%]	25 [47%]	152 [66%]	118 [47%]	0.413	0.007	<0.001
Pain in muscle, bones and joints	36 [58%]	36 [68%]	157 [69%]	164 [66%]	0.837	0.066	0.001
Chronic pain	23 [37%]	10 [19%]	115 [50%]	89 [36%]	0.011	0.043	<0.001
Fibromyalgia	4 [7%]	1 [2%]	45 [20%]	14 [6%]	0.183	0.011	<0.001
Chronic headache	20 [32%]	9 [17%]	88 [38%]	3 [27%]	0.026	0.293	<0.001
Dyspepsia	12 [19%]	3 [6%]	47 [21%]	17 [7%]	0.016	0.731	<0.001

Men with IBS rated their health lower than men without IBS (*p* = 0.002) and similarly women with IBS estimated their health lower than women without IBS (*p* < 0.001). The difference between men and women with IBS in terms of general health rating was not statistically significant (*p* = 0.927).

Despite the differences in health rate, health was equally important to the participants regardless of IBS diagnosis and sex/gender. Over 90% of the participants, regardless of IBS diagnosis, were actively or to some extent engaged in activities to promote their health. Comparison of men and women with IBS revealed a statistically significant difference in their activities in terms of health improvement (*p* = 0.037). For example, 8% of men with IBS compared to 3% of women with IBS made no efforts at all to improve their health. However, a similar men/women pattern was also seen in the control groups.

People with IBS reported a higher number of comorbidities than people without IBS. The following comorbidities dominated in men with IBS in comparison with men without IBS: chronic pain (*p* = 0.011), chronic headache (*p* = 0.026), and dyspepsia (*p* = 0.016). Compared to women without IBS, women with IBS more commonly suffered from psychiatric disorders (*p* < 0.001), sleep disorders (*p* < 0.001), fibromyalgia (*p* < 0.001), pain in muscles, bones, and joints (*p* = 0.001), or dyspepsia (*p* = 0.016). Men with IBS compared to women with IBS tend to have less chronic pain (*p* = 0.043), less psychiatric disorders (*p* = 0.027), and less sleep disorders and fibromyalgia (*p* = 0.011). The differences in chronic headache, pain in muscles, bones and joints, and dyspepsia between men and women with IBS were not statistically significant.

Analysis of contact with health care during the past 12 months revealed that there was no statistically significant difference between men with and without IBS as regard seeking health care (*p* = 0.914). At the same time, women with IBS have more contact with health care than women without IBS (*p* < 0.001) and men with IBS (*p* < 0.001).

### 3.3 Sex/gender differences in symptomatology

The sex/gender differences in IBS symptoms are shown in Table 3.

**TABLE 3:** Sex/gender differences in IBS symptomology

Variable	Men with IBS [*n*]	Men without IBS [*n*]	Women with IBS [*n*]	Women without IBS [*n*]	Men with IBS vs. men without IBS, *p*	Men with IBS vs. women with IBS, *p*	Women with IBS vs. women without IBS, *p*
Nausea							
Episodes/week	0.5	0	1	0	<0.0001	0.517	<0.001
Hours/week	0.5	0	1.8	0	<0.001	<0.001	<0.001
Abdominal pain							
Episodes/week	3	0	3	0	<0.001	0.613	<0.001
Hours/week	6.5	0	7.5	0	<0.001	0.954	<0.001
Light intensity, Episodes/week	0.4	0.3	0.4	0.5	0.702	0.875	0.422
Light intensity, Hours/week	1.1	0.67	1.0	0.69	0.063	0.356	0.193
Moderate and intense intensity, Episodes/week	0.4	0.3	0.5	0.5	0.694	0.686	0.113
Moderate and intense intensity, Hours/week	1.3	0.67	1	0.25	0.264	0.984	0.013
Left upper quadrant, Episodes/week	0.5	<0	0.5	<0	<0.0001	0.494	<0.001
Right upper quadrant, Episodes/week	0.5	<0	0.5	<0	<0.001	0.468	<0.001
Left lower quadrant, Episodes/week	1	<0	1.5	<0	<0.001	0.114	<0.001
Right lower quadrant, Episodes/week	1	<0	1.5	<0	<0.001	0.094	<0.001
Whole abdomen, Episodes/week	<0	<0	<0	<0	0.001	0.088	<0.001
Bloating							
Episodes/week	4	0	4	0	<0.001	0.836	<0.001
Hours/week	19	0	14	0	<0.001	0.836	<0.001
Defecations							
Stools, number/week	12	9	10	9	<0.001	0.034	<0.001
Bristol stool scale, cases per week							
Type 1	0.5	0	0.5	0	<0.001	0.438	<0.001
Type 2	0.5	0.5	0.5	0.5	0.098	0.705	0.495
Type 3	0.5	1	0.5	1	0.392	0.834	0.058
Type 4	2	2	1.5	2.5	0.148	0.934	<0.001
Type 5	2	1.5	2	1	0.289	0.404	<0.001
Type 6	1	0	0.5	0	<0.001	0.188	<0.001
Type 7	0.5	0	0	0	0.002	0.419	<0.001
Urgency, % of all defections	19	6	33	7	<0.001	0.017	<0.001
Straining, % of all defections	31	14	40	25	0.041	0.133	<0.001
Incomplete emptying, % of all defections	42	7	47	9	<0.001	0.146	<0.001

Nausea was statistically significant associated with having IBS. The difference in average number of nausea episodes per week was statistically significant when comparing men with and without IBS (*p* < 0.001), and women with and without IBS (*p* < 0.001). Men with IBS averaged one nausea episode per 2 weeks, while women with IBS had one episode of nausea per week (*p* = 0.517). An average duration of nausea per week was 0.5 h in men with IBS and 1.8 h in women with IBS (*p* < 0.001).

Analysis of episodes and duration of abdominal pain showed that both men and women with IBS had an average of 3 abdominal pain episodes per week. The average duration of abdominal pain was 6.5 h for men with IBS and 7.5 h for women with IBS (*p* = 0.954). No statistically significant differences were found in abdominal pain severity and duration between men and women with IBS. Pain localization between men and women with IBS was not statistically significant.

Patients with IBS, both men and women, had more episodes of bloating and longer duration of bloating compared to men and women without IBS. Men and women with IBS had on average 4 episodes per week (*p* = 0.836). Duration of bloating was on average 19 h per week in men with IBS and 15 h per week in women with IBS (*p* = 0.836).

There was a higher number of stools in patients with IBS compared to controls for both men (*p* < 0.001) and women (*p* < 0.001). The number of stools was on average 12 and 10 per week in men and women with IBS, respectively (*p* = 0.034). The difference in stool consistency in men and women with IBS was not statistically significant.

Urgency was more prevalent in men and women with IBS than in the controls (*p* < 0.001 in both cases). Women with IBS had a higher percentage of defecations with urgency than men with IBS: 33% and 19% of all defecations, respectively (*p* = 0.017). Straining in relation to defecation was also more common in men (*p* = 0.041) and women (*p* < 0.001) with IBS, compared to the controls. Men with IBS had to strain on average in 31% of the defecations; for women with IBS, this figure was 40% (*p* = 0.133). The same pattern was seen when comparing defecations with the feeling of incomplete emptying. It was more common for people with IBS, both men and women, to have defecations with the feeling of incomplete emptying compared to the controls (*p* < 0.001 in both cases). Men with IBS had on average 42% of defecations with incomplete emptying, compared to 47% for women with IBS (*p* = 0.146).

## 4 DISCUSSION

The main findings were that (1) there was no difference between men with and without IBS in terms of educational level, satisfaction with household economy, or living with a partner. In contrast, women with IBS more often lived alone, were more often dissatisfied with household economy, and had a lower educational level than women without IBS; (2) men with IBS had the same proportion of full‐time employment as men without IBS, but in contrast, the proportion of women with IBS working full‐time was only 34% compared to 50% of the women without IBS; (3) men with IBS reported a lower degree of activity compared to women with IBS when it comes to health‐improving activities; (4) men with IBS, when compared to women with IBS, had less contact with the healthcare system, fewer psychiatric comorbidities, fewer sleeping problems, and less chronic pain in general, but a similar degree of abdominal pain severity, frequency, and location; and (5) the only gastrointestinal symptoms that differed between men and women with IBS were urgency to defecate and nausea, which were less common in men, and stool frequency, which was higher in men with IBS.

### 4.1 Sex/gender differences in terms of health/disease status

Women with IBS had more contact with health care than women without IBS and men with IBS. Men are less likely to utilize healthcare services than women are, even when they have access to these services.^23^, ^24^ Some of the potential explanations for “under‐usage” of medical care by men relate to structural organization of the healthcare system, work conditions, lack of experience of seeking health care, and lack of experience of talking about their own health problems, as well as traditional notions of masculinity. Insufficient information about men's health issues and the absence of health programs targeted at men contribute to lower awareness of specific health problems or symptoms in men, and consequently less likelihood to seek medical help and have regular health controls.^23^ The association of masculinity with physical strength, control, and stoicism contributes to viewing healthcare‐seeking behavior as a personal failure, an expression of weakness and a threat to masculinity. This might result in men's lack of a vocabulary for talking about sensitive issues, and an inability to express weakness, discuss vulnerability, and reveal pain.^24^, ^25^ This in its turn might discourage them from disclosing physical and psychological symptoms, including those relevant to IBS and seeking health care.

The difference between men and women with IBS in terms of general health rating was not statistically significant. Other studies have shown that men with IBS tend to have lower levels of fatigue, more positive well‐being and better self‐control than women with IBS.^5^ They have also higher IBS QoL (quality of life) scores, higher satisfaction with bowels habits and higher body image.^5^ However, we found that men with IBS rated their health lower than men without IBS, and similarly, women with IBS estimated their health lower than women without IBS. This finding is most likely connected with both the presence of IBS symptoms and higher comorbidity burden in people with IBS. Our study also revealed that men with IBS have fewer comorbidities than women with IBS, which can contribute to men's lower rate of seeking health care.

Based on earlier analyses of a similar study population, the IBS patients were less likely to report “good” health status and were also less likely to report a positive belief in the future, in comparison with the controls without IBS.^26^ There was also indication that the IBS cases on average scored higher on the negative self‐esteem measure and lower on the positive self‐esteem measure than controls^26^ which potentially could be associated with sex differences, too, since men with IBS in earlier studies demonstrated higher levels of SOC, compared to women with IBS.^11^, ^27^, ^28^

Men with IBS had the same proportion of full‐time employment as men without IBS (63% vs. 61%). In contrast, the proportion of women with IBS working full‐time was only 34%, compared to 50% of the women without IBS. Gendered systems of inequalities contribute to higher level of chronic stress factors and higher IBS vulnerability of women. This relates to lower access to economic resources, lower salaries, unpaid household work, women's nurturing roles, and violence against women.^12^ Men with IBS also experience stress; however, it is more often connected with negative influence of IBS symptoms on their paid employment, responsibility for family finances, career opportunities, and sense of control.^10^ However, this study demonstrates that men with IBS have significantly better employment status than women with IBS, and that their employment status does not differ from that of men without IBS. This in turn indicates that men with IBS experience more favorable work opportunities than women with IBS. This can partially reflect gender inequalities in paid and unpaid work in society, and can be related to gender differences in IBS symptoms and associated comorbidities.

### 4.2 Sex/gender differences and comorbidities

As seen in our previous study among of cohort, all IBS patients had more comorbidities compared to the non‐IBS patients,^20^ and surprisingly, the number of comorbidities was not a predictor of reported good health in either IBS cases or in the control group without IBS. Although the present study did not discuss sex/gender differences concerning the total comorbidity burden, it revealed that men with IBS have fewer comorbidities than women with IBS. When focusing on sex differences, this study showed that men with IBS tend to have less chronic pain, fewer psychiatric disorders, fewer sleep disorders, and less fibromyalgia than women with IBS. Men with IBS also have less chronic headache, less pain in muscles, bones, and joints, and less dyspepsia than women with IBS, although the differences are not statistically significant. This demonstrates that despite similarities in IBS symptomology, men with IBS have fewer comorbidities than women with IBS.

The relationships between the psychological symptoms and IBS can be bidirectional. Psychological factors may affect and modulate patients' perception of their illness, and IBS symptoms can have a negative influence on psychological health.^29^ There is an inconsistency in evidence in sex/gender differences in psychological symptoms in patients with IBS. Some studies show a higher prevalence of anxiety and depression in women with IBS,^13^ while others demonstrate no such sex/gender difference.^30^, ^31^ According to the study by Björkman, women report higher general anxiety, but no differences between men and women are found as to depression.^11^ This study supports evidence for the overall higher prevalence of psychiatric disorders in women with IBS compared to men with IBS, regardless of specific psychological conditions.

### 4.3 Gastrointestinal symptoms in men and women with IBS

Urgency is the unpleasant sensation that makes one rush to the toilet to avoid leakage. In the present study, urgency was less often reported by men with IBS than by women with IBS. Sex differences in terms of urgency to defecate have also been reported in the general population, with a female predominance.^32^ Urgency is often associated with loose stools and diarrhea, but in IBS it is frequently also present in relation to normal or hard stool consistency.^33^ A recent study found that urgency prior to anal leakage was more prevalent in women than in men, and in the same study, IBS was found to be a risk factor for anal incontinence.^34^

The two other defecatory symptoms that were registered—the need to strain during defecation and the feeling of incomplete evacuation—were similar in women and men with IBS. To our knowledge, these defecatory symptoms have not previously been compared between men and women with IBS. Perveen et al. found no difference in defecatory symptoms in females and males with functional constipation.^35^ Nor did Walter et al. when in a population‐based study they compared more than 250 randomly selected subjects. However, females had significantly more symptoms than males in terms of the feeling of incomplete evacuation as well as urgency, before excluding patients with IBS.^36^ Frequency of defecation varies widely in healthy adults but is mostly reported to be between three times per day and three times per week in both sexes.^36^, ^37^, ^38^ In this study, men with IBS had more bowel movements per week than women with IBS, which was an expected finding, since men in general are known to have a shorter colonic transit time than women.^39^

There were no statistically differences between men and women with IBS in terms of abdominal pain severity, duration, or localization. These findings are in contrast to other studies which discuss lower vulnerability of men for abdominal pain sensitivity.^9^, ^15^ The role of sex hormones in IBS is mentioned as an important aspect of sex difference in the experience of IBS. Men unlike women do not experience a significant IBS symptoms variation or intensification of GI symptoms, such as bloating, abdominal pain, and intestinal gas, related to hormone variations in different menstrual phases or during menopause.^15^ Male gonadal hormones might have a protective role for IBS development and symptom representation in men.^9^ Testosterone in particular might have an analgesic effect by decreasing pain sensitivity and protecting against chronic pain disorders.^15^ Difference in response to intestinal stimuli and pain perception in men and women can also be linked to morphological and functional brain differences.^40^ The differences between the results of these studies could partly be explained by the fact that this study does not focus on symptoms' variation in women related to menstrual cycle. Thus, the data reflect only the symptoms during the 2 weeks of the study period, with no relation to a potential intensification of pain and other symptoms in women in relation to menstruation. Another reason for difference in the results is that different methods have been used to measure pain. In experimental settings, pain thresholds and pain levels are often related to a specific stimulus rather than to the self‐report diaries and questionnaires that were used in our study. The latter might be more favorable to men, because men might be more open to reporting their experience of pain in self‐reported measurements as they might feel less social pressure to express physical strength and stoicism in front of others, which could be the case in experimental settings.^8^

The frequency of bloating was also similar in men and women with IBS. This is in line with the findings of Kosako et al. in the subgroup IBS‐C.^40^ However, Jiang et al. found that female sex increased the risk of distension over bloating alone.^41^

A strength of the present study is that validated symptom diaries were used. Recall has been studied across a range of disease states and the time interval during which events are recalled has been shown to be relevant to recall accuracy both in general^42^ and in gastrointestinal disease. The concordance between gastrointestinal symptoms when measured by questionnaire versus diary has been poor in several studies.^43^, ^44^, ^45^ A possible limitation of using IBS diagnoses made at a PHC, as done in the present study, is the dependence on the general practitioners' ability to make the correct diagnosis. On the contrary, it could also be a strength, because most IBS patients are diagnosed in primary care. Another limitation is that the absence of Gaussian distribution in a subset of our data may reflect a variety of biases. On the contrary, there is the strength are that our selection of a control group, which in this case consists of patients without GI problems, enables us to find associations to IBS itself. In contrast to many other studies in the field, this research allows multiple comparisons. This in turns allows a more detailed examination of not only differences between men and women with IBS, but also between men with and without IBS.

## 5 CONCLUSION

Sex and gender intersect with all aspects of IBS—pathophysiology (early life/environment, psychological factors, physiology), presentation (symptoms, severity, behaviors), and outcomes (healthcare use, daily function, quality of life, healthcare costs). Sex and gender have not been explicitly integrated into the biopsychosocial model of IBS. However, they are crucial for understanding the differences in individual experiences of the disease, and cannot be omitted in a discussion of IBS's etiology, presentations, and treatment strategies. Despite the growing interest in sex/gender dimensions of IBS in recent years, the predominant focus has been on women's experiences. Our study attempts to contribute to a better understanding of men's experiences of IBS, and suggests a potential explanation of the significant gender differences in healthcare‐seeking behavior and prevalence of IBS.

## AUTHOR CONTRIBUTIONS

ÅF, SW, and AKN involved in concept and design. ÅF, SW, AKN, and JS involved in acquisition of data. TB, ÅF, SW, and AKN involved in analysis and interpretation of data. TB, ÅF, AKN, SW, and JS involved in drafting of manuscript. TB, ÅF, SW, JS, and AKN involved in critical revision of manuscript for important intellectual content. TB involved in statistical analysis. All authors read and approved the final manuscript.

## FUNDING INFORMATION

This study was supported by FORSS—the Research Council of Southeast, Sweden.

## DISCLOSURE

The authors have no competing interests.
